# The association between seizures and deposition of collagen in the brain in porcine *Taenia solium* neurocysticercosis

**DOI:** 10.1016/j.vetpar.2016.09.008

**Published:** 2016-09-15

**Authors:** Nina M. Christensen, Chiara Trevisan, Páll S. Leifsson, Maria V. Johansen

**Affiliations:** Department of Veterinary Disease Biology, Faculty of Health and Medical Sciences, University of Copenhagen, Dyrlægevej 100, Frederiksberg C, Denmark

**Keywords:** Histopathology, Epilepsy, Pigs, Brain tissue, Infection

## Abstract

Neurocysticercosis caused by infection with *Taenia solium* is a significant cause of epilepsy and seizures in humans. The aim of this study was to assess the association between seizures and the deposition of collagen in brain tissue in pigs with *T. solium* neurocysticercosis. In total 78 brain tissue sections from seven pigs were examined histopathologically i.e. two pigs with epileptic seizures and *T. solium* cysts, four pigs without seizures but with cysts, and one non-infected control pig.

Pigs with epileptic seizures had a larger amount of collagen in their brain tissue, showing as large fibrotic scars and moderate amount of collagen deposited around cysts, compared to pigs without seizures and the negative control pig. Our results indicate that collagen is likely to play a considerable part in the pathogenesis of seizures in *T. solium* neurocysticercosis.

## Highlights

• Pigs with seizures had larger amount of collagen compared to pigs without seizures.
• Collagen is likely to play a role in the pathogenesis of seizures in neurocysticercosis.
• Pigs can serve as a model for studying the pathogenesis of seizures in neurocysticercosis.

## Graphical abstract

**Figure:**
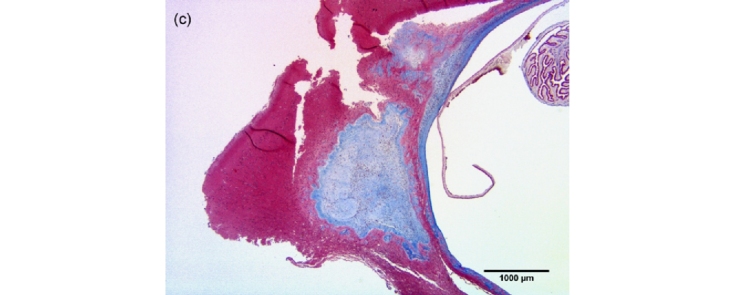

## 1 Introduction

*Taenia solium* is the causative agent of neurocysticercosis in humans and pigs due to lodging of cysticerci in the central nervous system. Epileptic seizures account for the majority of clinical manifestations in human *T. solium* neurocysticercosis (Garcia et al., 2003). Results of a systematic review suggested that a diagnosis of neurocysticercosis could be made in nearly one-third of epilepsy cases in areas where *T. solium* is endemic (Ndimubanzi et al., 2010). However, a significant proportion of individuals with neurocysticercosis remain asymptomatic (de Almeida and Torres, 2011), which may partly be ascribed to the fact that it can take years from infection time until signs start showing (Garcia et al., 2003). Recently, Trevisan et al. (2016) reported for the first time seizures in pigs with *T. solium* neurocysticercosis (Trevisan et al., 2016). Previous to this, clinical signs in pigs have not been studied thoroughly, and pigs have to a large extent been considered not to be clinically affected by the disease. What triggers the development of seizures in *T. solium* neurocysticercosis is not clearly determined. A local inflammatory response evoked by antigen release from degenerating cysticerci has been associated with seizure development, but the degree of inflammation cannot predict the clinical outcome in all cases. Besides the inflammatory response, deposition of collagen type I around brain cysticerci has been described in both human and porcine specimens (Alvarez et al., 2002, Restrepo et al., 2001). The aim of our study was to assess the association between seizures and the deposition of collagen in brain tissue in pigs with *T. solium* neurocysticercosis.

## 2 Materials and methods

Brain tissue was obtained from six pigs from Kongwa district, Dodoma region, Tanzania. These were naturally infected with *T. solium*, and diagnosis was based on tongue inspection. The infected pigs were part of an experimental behavioral study conducted over 14 consecutive days and in which seizures were observed in two out of 16 pigs (Trevisan et al., 2016). Two infected pigs with seizures and *T. solium* cysts, four infected pigs without seizures but with cysts and one non-infected control pig originating from a commercial Danish pig farm, were included in the present study. Among the 14 pigs without seizures, two pigs were excluded from selection due to nonspecific disease signs during the experimental behavioral study. Of the remaining 12 pigs, the four controls were selected based on the total number of cysts in the brain found by necropsy, selecting seizure-free pigs with the highest number of cysts. The total number of brain cysts in these four pigs were 418, 117, 90, and 88, respectively. The total number of brain cysts in the two pigs with seizures were 247 and 241, respectively. Based on their dentition, the estimated age of the two pigs with seizures was 36 months, while the estimated age of the four pigs without seizures was between 12 and 24 months. Following necropsy, brain tissue specimens of approximately 1 cm^3^ were cut out and fixed in 10% neutral buffered formalin for minimum 24 h and subsequently paraffin-embedded. From each infected pig and each brain hemisphere, two histological tissue sections of 1–2 μm thickness were made from the frontal, temporal, and occipital lobe. Sections were deparaffinised, rehydrated and stained. Of the two sections representing the same region of the brain, one section was stained with haematoxylin and eosin (HE) and the other with Masson’s trichrome using Weigert’s iron haemtoxylin, Biebrich scarlet acid fuchsin, and methyl blue for the detection of collagen (Bancroft and Gamble, 2008).

As a reference for normal porcine brain tissue, six histological sections were made from brain tissue specimens from the non-infected pig according to the method described above, but only from one brain hemisphere. Paraffin-embedded brain tissue specimens from this pig were provided by Section for Experimental Animal Models, Department of Veterinary Disease Biology, University of Copenhagen, Denmark.

In total 78 histological sections of brain tissue were examined by histopathological examination using a light microscope (Leica DM LB). Blue staining of tissue was interpreted as collagen-positive. The amount of collagen deposition in relation to individual cysts was assessed by visual examination and categorized as: no to minimal, moderate or large amount. The association between seizures and the amount of collagen was tested statistically with a chi-square test.

### 2.1 Ethical clearance

All procedures employed in the study were approved by Sokoine University of Agriculture, Morogoro, Tanzania (Ref. no. RPGS/R/AS/42/2014) and in accordance with the national guidelines of ethics for health research and to the Animal welfare act (2008) (Mashalla et al., 2009, The United Republic of Tanzania, 2008).

## 3 Results

In total 78 histological sections of brain tissue were examined. From the *T. solium* infected pigs 60 cysticercal lesions were identified. In 57 of them, the vesicular membrane of the parasite was visible. Three lesions, all observed on sections from one of the two pigs with seizures, appeared as large demarcated fibrotic areas and categorized as having a large amount of collagen. These lesions were interpreted as scars after complete degradation of the cysticerci. No parasite structures were observed within these lesions. The fibrotic scars were characterized by compact deposition of collagen staining densely blue. Few inflammatory cells including mononuclear cells and eosinophils were interspersed between the fibroblasts and collagen fibers. Signs of a previous process of necrosis (malacia) were evident in one of the fibrotic scars. This displayed as a central cavity in the middle of the fibrotic area in which phagocytising macrophages with intracytoplasmic debris-like material and collagen strands forming a mesh like structure were observed.

The cysticerci with visible parasite structures were surrounded by no to a minimal amount or by a moderate amount of collagen. In addition, various degrees of inflammation surrounded the cysticerci. All cysticerci (22 out of 22) examined from the pigs with seizures were surrounded by a moderate amount of collagen. In pigs without seizures, nine out of 35 cysticerci (25.8%) were surrounded by no to a minimal amount of collagen. The remaining 26 out of 35 cysticerci (74.2%) in pigs without seizures were surrounded by a moderate amount of collagen. The difference in amount of collagen between pigs with and without seizures was significant (*P* = 0.012).

Collagen in the brain tissue from the non-infected pig was observed only as very thin strands in relation to blood vessels and meninges.

## 4 Discussion

This is the first study that describes histopathological findings in the brain tissue of pigs with neurocysticercosis and concomitant seizures. The overall histopathological findings with respect to deposition of collagen were to an extent different between pigs with and without seizures. Large fibrotic scars observed in one pig with seizures and absence of no or minimal amount of collagen around a proportion of cysticerci in pigs without seizures accounted for this difference. These findings indicate that the deposition of collagen in the brain and seizures in porcine *T. solium* neurocysticercosis are possibly associated variables. An association between seizures and the larger amount of collagen could furthermore point to a causal relationship, suggesting an important role of collagen in the pathogenesis of seizures in *T. solium* neurocysticercosis. The role of cortical deposition of collagen in posttraumatic epilepsy was proposed almost a century ago by Penfield (1927). Furthermore, Hoeppner and Morrell (1986) demonstrated that epileptic activity provoked by experimentally induced trauma to the cerebral cortex of guinea pigs could be reduced by using methods that inhibited collagenous scar formation (Hoeppner and Morrell, 1986). The mechanism behind an epileptogenic effect of collagen has been suggested to result from a contracting scar leading to chronic irritation and eventually increased neuronal discharge (Penfield, 1927). Based on the substantial deposition of collagen it is obvious to consider that the same mechanism could be implicated in *T. solium* neurocysticercosis. If so, it would also explain the long incubation time of the disease seen in many cases of human neurocysticercosis.

It is likely that the larger amount of collagen observed in the pigs with seizures was due to longer duration of infection in these pigs. This is supported by the fact that the pigs with seizures were between 12 and 24 months older than the pigs without seizures, as the age of infection is presumably more likely to be higher in older compared to younger individuals (Garcia et al., 2003).

As only selected regions of brain tissue were studied, it cannot be discarded that similar large fibrotic scars were also present in the brain tissue from other pigs. There is a need for further investigations on this area with larger study groups in order to confirm the findings of this study. The recent observations of seizures in pigs with this disease open up for new potentials for studying the pathogenesis of seizures in *T. solium* neurocysticercosis using the pig as a model.
